# Acknowledgement to Reviewers of *Entropy* in 2019

**DOI:** 10.3390/e22020130

**Published:** 2020-01-21

**Authors:** Entropy Editorial Office

**Affiliations:** MDPI, St. Alban-Anlage 66, 4052 Basel, Switzerland

The editorial team greatly appreciates the reviewers who have dedicated their considerable time and expertise to the journal’s rigorous editorial process over the past 12 months, regardless of whether the papers are finally published or not. In 2019, a total of 1274 papers were published in the journal, with a median time to first decision of 17.8 days and a median time from submission to publication of 2.7 days. The editors would like to express their sincere gratitude to the following reviewers for their generous contribution in 2019:
Abah, ObinnaLöbel, MartinAbbaschian, RezaLoewer, BarryAbd-Alhameed, RaedLöfstedt, TommyAbdel-Aty, MahmoudLoizos, AndreasAbdollahzadeh Jamalabadi, Mohamad YaghoubLombardi, OlimpiaAbel, AndrewLong, Gui-LuAbenza, Pedro Pablo GarridoLong, JunAbraham, EleniLongobardi, MariaAbramov, ViktorLopez-Corona, OliverAbreu, Everton M.C.López-Estrada, Francisco RonayAbu Shattal, MohammadLopez-Menendez, Ana JesusAbuzaid, WaelLopez-Monsalvo, Cesar S.Acedo Rodríguez, LuisLópez-Morales, VirgilioAcedo, LuisLorenz, RalphAcevedo, Rubén CarlosLorenzo, SalvatoreAcharya, U RajendraLosee, Robert M.Adamatzky, AndrewLostado, RubénAdams, StefanLoubenets, Elena R.Afify, AhmedLouzguine, Dmitri V.Agnew, BrianLovato, AlessandroAguirre-Hernández, BaltazarLove, Jeffrey J.Ahamed, NizamLoza, Carlos AAhammer, HelmutLozi, RenéAhmad, JawadLozi, RenéAhmadi, MohammadLu, ChaoAhmed, Mosabber UddinLu, ChenyangAkbari, MohammadLu, Chia-FengAkhmetzhanov, Andrei R.Lu, QinAkhundjanov, SherzodLu, QinghuaAlamaniotis, MiltiadisLu, RongxingAla-Nissila, TapioLu, YangAlava, MikkoLu, ZhenboAlbantakis, LarissaLu, ZhixinAlberti, TommasoLuca, AdrianAlbu, FelixLucia, UmbertoAlbuquerque, Holokx A.Łuczak, MaciejAlebraheem, JawdatŁuczka, JerzyAlejo Eleuterio, RobertoLudovisi, AlessandroAlexandridis, Antonio T.Lunin, OlegAlfonso, LeonardoLuo, AlbertAliakbarzadeh, MojtabaLuo, BinAlicea, BradlyLuo, Jia-NingAliyu, AliyuLuo, XiongAl-Jaber, Sami MohammadLuque, JoaquínAljankawey, Abdualah S.Luscuere, PeterAllen, RolandLuszczek, PiotrAlmeida, FernandoLuukka, PasiAlmeida, ThiagoLuzzi, LauraAlmgren, KhaledLymperis, AndreasAL-Naji, AliLyubushin, AlexeyAlodjants, AlexanderM. Hangos, KatalinAlonso, FabiolaMa, DandanAlonso, JoanMa, RuichaoAlonso, Miguel A.Ma, YongshengAlonso-Serrano, AnaMaaref, HichemAltaisky, M.V.Maasoumi, EsfandiarAltman, MicahMacaluso, AdrianoAltmann, EduardoMacBean, VictoriaÁlvarez Pardiñas, ÁngelMacci, ClaudioÁlvarez, María S.Macêdo, Antônio M.S.Alvarez-Hamelin, José IgnacioMachado, TenreiroAlvarez-Ramirez, JoseMachrafi, HatimAlves, LuizMaciag, ArturAlvim, Mário S.Maciejewski, WesAmancio, DiegoMaciel, Carlos D.Amezquita Sanchez, Juan PabloMacLaurin, James N.Amigó, José MaríaMaddamsetti, RohanAmini, M. HadiMadej, M. GregorAmosov, GrigoriMadiman, MokshayAnagnostopoulos, GeorgiosMadokoro, HirokazuAnand, VijayMady, Carlos Eduardo KeutenedjianAnastassopoulos, VassilisMaestre Vidal, JorgeAndersen, UlrikMagafas, LykourgosAndriukaitis, DariusMagnot, Jean-PierreAngel, LuisMagreñán, Ángel AlbertoAnghel, Dragos-VictorMahian, OmidAngulo, Juan CarlosMahlia, T M IndraAngus, SimonMahmoodi, KoroshAnishchenko, LesyaMai, FengAnnunziata, OnofrioMailybaev, AlexeiAnochi, JulianaMainprize, JamesAntoniou, MarkMaire, JeremieAntonopoulos, AngelosMajak, JüriAoyagi, KentaMajlinger, KornelApfelbaum, Evgeny M.Mak, Chi H.Apiecionek, ŁukaszMaki, KaraApollaro, Tony John GeorgeMakihara, KanjuroAqueveque, PabloMakinde, Oluwole D.Arampatzis, GeorgiosMakowiec, DanutaAraújo, DuarteMalecha, ZiemowitAravind, Padmanabhan K.Małecki, KrzysztofArbel, JulyanMálek, JaroslavArce, Pedro F.Malekian, RezaArdenghi, Juan SebastiánMalhis, NawarAres De Parga-Álvarez, GonzaloMalka, DrorArguelles-Cruz, Amadeo J.Malone, BrandonArima, TakashiMalvoni, MariaAristov, YuriMameli, ValentinaArizmendi, Constancio MiguelMan’ko, MargaritaArminen, IlkkaManavalan, BalachandranArnas, OzerMånberger, AndréAros, RodrigoMancini, StefanoArraut, IvanMancino, LucaArsac, Laurent M.Mandal, SubhamoyAryan, PouriaManis, GeorgeAsadi, AminManko, Vladimir I.Ascigil, OnurManzano, GonzaloAsikuzzaman, MdMao, YongAsoodeh, ShahabMarano, MassimoAsorey, ManuelMarateb, Hamid RezaAsorey-Cacheda, RafaelMarchildon, LouisAssunção, PedroMarchioli, CristianAtanassov, KrassimirMarcon, EricAtilhan, MertMardani, AbbasAtman, AllbensMardziel, PiotrAtoche, Alejandro CastilloMargot, XandraAttili, AntonioMari, FedericoAurelian, IsarMARIASIU, FlorinAusloos, MarcelMarinazzo, DanieleÁvalos, Juan GerardoMarino, ArmandoAvanaki, KamranMarjanovic, MarinaAvanaki, Mohammad R.N.Markovič, ReneAvila, Sergio LucianoMarkussen, BoAwad, Mohamed M.Marolt, MatijaAxinte, EugenMarović, IvanAydin, ÜmitMarsiglietti, ArnaudAzab, MohamedMartian, AlexandruAzami, HamedMartin, James D.Aziz, MuhammadMartin, LuciAziz, WajidMartín-Aragón, SagrarioAzzedin, FaragMartín-Clemente, RubénBaars, Woutijn J.Martinez Rodriguez, FlemingBabichev, SergiiMartínez-Val, J.M.Bacanin, NebojsaMartyushev, LeonidBaccala, Luiz AntonioMarwan, NorbertBacco, DavideMarzen, SarahBadcock, PaulMarzolino, UgoBader, Brett W.Masiello, AntonioBadin, GualtieroMassari, FilippoBadur, JanuszMassari, FrancescoBaena, Francisco ManuelMastrangeli, MassimoBagchi, SusmitMastriani, MarioBahamonde, SebastiánMatei, AniBai, BofengMateos, CristianBaicheva, TsonkaMatera, MauricioBailón, RaquelMathewson, Kyle ElliottBaingana, BrianMathias, Mauro HugoBajic, DraganaMatsoukas, ThemisBakar, ShuvoMatsumoto, RyutarohBaker, IanMatsuno, KoichiroBaker, TharMatsushima, ToshiyasuBakhtin, VictorMatus, MedoBalabdaoui, FadouaMaudlin, TimBalasis, GeorgeMaunder, RobBaldominos, AlejandroMayer, ChristopherBaleta, JakovMazurek, PrzemysławBaležentis, TomasMazzei, LorenzoBalk, Alexander M.Mazzulla, GabriellaBall, AureliaMcAssey, EdwardBallesteros, Dora M.McDonough, Ian M.Balthazar, JoseMcGarr, Arthur F.Bamba, KazuharuMcGuire, MichaelBan, MasashiMeade, AidanBanawan, KarimMebarek Oudina, FatehBandt, ChristophMeda, AliceBania, PiotrMedellín-Rodríguez, Francisco J.Banisch, RalfMedica-Viola, VedranBaranovskii, Evgenii S.Medrano, MarcBarbieri, MarcoMegías Jiménez, DavidBarbosa, RommelMehmood, FaizanBarddal, Jean PaulMeidute-Kavaliauskiene, IevaBariviera, Aurelio FernandezMeier, Robert J.Barra, FelipeMeis, ConstantinBarradas, Nuno PessoaMejía-Monasterio, CarlosBarranco-Jiménez, MarcoMelbourne, James C.Barrett, AdamMelie Garcia, LesterBarrio, RobertoMelle-Franco, ManuelBarros, Julio M.Melloni, LuciaBartlett, Stuart Melnikov, Alexander S.Başağaoğlu, HakanMemon, ImranBasak, SujitMenasalvas, ErnestinaBasaran, CemalMéndez, VicençBaselice, FabioMendez-Bermudez, J.A.Basieva, IrinaMenéndez, Héctor D.Basios, VasileiosMeng, GuozhuBasnarkov, LaskoMeng, TianhuiBastiaans, RobMentel, GrzegorzBatagin-Neto, AugustoMeo, MariannaBaudot, PierreMerchán, PilarBaugh, JonathanMercorelli, PaoloBaxter, GarethMerelli, EmanuelaBeal, AubreyMesa Sanchez, Oscar JoseBeau, MathieuMessina, NinniBecerra, Miguel A.Miani, Rodrigo SanchesBecerra, VictorMiao, QingfengBeckstead, JasonMichalski, RadoslawBeckwith, AndrewMichelini, FabienneBekiros, SteliosMiettinen, JariBelaid, AbdelMigdalas, AthanasiosBelman-Flores, J.M.Mihailovic, DragutinBelonenko, M.B.Milanesi, PietroBelyaev, EvgenyMilani, AlfredoBelyi, AlexanderMillen, Jonathan K.Ben Naim, AriehMiller, BorisBen Sherwood, BenMillington, PeterBenavente, CésarMimoso, José PedroBenedic, FabienMinak, GiangiacomoBenenti, GiulianoMinati, Hab. LudovicoBenet, LuisMinea, Alina AdrianaBenito, Rosa M.Minussi, Carlos RobertoBenito, SoniaMiramontes, OctavioBentes, CarlosMiramonti, LinoBentsman, JosephMiret-Artés, SalvadorBeranek, LadislavMishra, AashwinBergstra, Jan AldertMishura, YuliyaBernaś, MarcinMisirlis, DimitriosBernhauerová, VeronikaMiskiewicz, JanuszBernstein, JosephMitchell, Paul DanielBerrett, ThomasMitrovic, NikolaBerry, TyrusModanese, GiovanniBerthon, ChristopheModig, KristoferBerthouze, LucMohaisen, ManarBessa, Iury ValenteMohammed, Hussein A.Besteiro, Lucas VazquezMoita, Ana SofiaBhattacharjee, SubrataMondejar, Maria E.Bhattacharya, TanmoyMonica, CosteaBhatti, M.M.Monteiro Santos, FernandoBhowmik, DebsindhuMontévil, MaëlBiagi, Pier FrancescoMontiel, OscarBialynicki-Birula, IwoMontoliu, RaúlBianchi, AlessandroMontoya-Castillo, AndrésBianconi, FrancescoMookiah, Muthu Rama KrishnanBianconi, GinestraMoon, KyuminBica, MarianMoore, Talia Y.Bicer, YusufMoosa, MudassirBier, MartinMorabito, Francesco CarloBietenholz, Wolfgang PeterMorales-Luna, GuillermoBiferale, LucaMoraru, LuminiţaBikov, AndrásMoravcik, IgorBilotta, GiuseppeMorchid, MohamedBiscarini, ChiaraMoreno, JaimeBitbol, MichelMoreno, Javier CerrilloBizarro, João P.S.Moreno-Valenzuela, JavierBlakely, JonathanMorgado, Welles Antonio MartinezBlanco Mesa, Fabio RaulMoriya, HirokazuBlanco, IgnazioMorley, BruceBlanes, IanMoroni, DavideBlecich, PaoloMorosuk, TatianaBlokhin, Alexander M.Morrison, GregBlokhuis, Edgar M.Moshrefi-Torbati, MohamedBlossey, RalfMoszkowski, StevenBlum, AlexanderMousavi, AliBlusseau, SamyMoussas, XenophonBobulski, JanuszMporas, IosifBocharova, Irina E.Mraz, MihaBode, FlorinMück, WolfgangBodini, AntonellaMueller, DavidBoffetta, GuidoMühlhaus, TimoBognár, GabriellaMujahid, Syed N.Bohdalová, MáriaMukherjee, PritamBojanic, Antonio N.Muller, IngoBolboacă, Sorana D.Muncharaz, Javier OliverBollt, Erik M.Munoz-Pacheco, Jesus M.Bolognesi, TommasoMuntendam-Bos, AnnemarieBonanno, ClaudioMurata, NoboruBonchev, DanailMuratore-Ginanneschi, PaoloBonilla, Luis L.Murcio-Villanueva, RobertoBonnal, PierreMurillo-Escobar, MiguelBoon, Jean PierreMurphy, BrendanBordoni, MassimilianoMurugan, N. ArulBorges Sebastião, IsraelMuschik, WolfgangBorgonovi, FaustoMuthuswamy, BharathwajBorgonovo, EmanueleMuzyk, MarekBorissov, Yuri L.Mwesigye, AggreyBorland, LisaMylonas, PhivosBormashenko, EdwardCirillo, Emilio N.M.Bošnjaković, MladenNadais, HelenaBossé, ÉloiNagy, SzilviaBostenaru Dan, MariaNaik, Ganesh R.Boström, KimNakajima, ToshiyukiBottazzi, GiulioNakamura, MakotoBouchard, KevinNakamura, RyotaBoudjelal, AbdelwahhabNakamura, Tadas K.Boughn, StephenNakonieczny, ŁukaszBoukas, AndreasNalepa, JakubBoulouz, AbdellahNam, KwanghoBousfield, Douglas W.Namazi, HamidrezaBouyuklieva, StefkaNapa, Sae-BaeBoyd, AlexanderNappi, CiroBoyer, Pierre C.Náprstek, JiříBradley, ChrisNarayan, Darren A.Braggio, AlessandroNarayanan, RamBranciard, CyrilNardelli, GiuseppeBravetti, AlessandroNardi, FernandoBredikhin, VladimirNarducci, DarioBremer, JörgNaseer, NomanBrevik, Iver H.Nashed, Youssef S.G.Brinck, ToreNaudts, JanBrinkworth, RussellNavara, MirkoBrinton, Christopher G.Navarrete, MiguelBross, ShragaNavarro-Guerrero, NicolásBrown, MichaelNayar, PiotrBrumen, BoštjanNazarov, Yuli V.Bruschi, David EdwardNazeer, MuhammadBudhathoki, KailashNeagu, GabrielBudzan, SebastianNedjalkov, MihailBuiu, CatalinNeilsen, Mitchell L.Bukovsky, IvoNejadsattari, FarshadBurin, Alexander L.Nelson, KenricBurns, StephenNene, SaurabhBurr, AlisterNepomuceno, Erivelton GeraldoBurrows, John P.Neri, IzaakBustos Marún, Raúl AlbertoNeto, Omar Paranaiba VilelaBustos, Diego MartinNetto, Vinicius M.Buszko, KatarzynaNeuman, YairButusov, DenisNeves, Leandro A.Buysschaert, JoostNeymotin, SamGeiger, Bernhard C.Ngamsombat, ChanonCabaleiro, DavidNguyen, BinhCabello, AdanNguyen, DinhCabessa, JérémieNguyen, VuCacciatori, SergioNguyen-Manh, DucCaesarendra, WahyuNiazi, Imran KhanCafagna, DonatoNicholson, SchuylerCafaro, CarloNicolaou, GeorgiosCagnoni, StefanoNicolaou, Nicoletta (Cyprus)Cai, BaopingNicolaou, Nicoletta (UK)Caines, Peter E.Nieto, JuanCaleffi, MarcelloNieuwenhuizen, TheoCalif, RudyNiezgoda-Zelazko, BeataCaligiuri, Michael P.Nikolova, NataliaCamarena-Martinez, DavidNikulov, AlexeyCamargo, ChicoNina, AleksandraCambou, BertrandNing, WeiCamci, CengizNiño-Ruiz, Elias DavidCamiz, SergioNiranjan, MahesanCampbell, JohnNiro, AlfonsoCampbell, SteveNisar, Kottakkaran SooppyCampisi, TizianaNita, AndreeaCampo Vázquez, CelesteNiu, KaiCamporeale, Sergio M.Niu, YulingCampos Souza, Paulo VitorNiven, RobertCandido, RenatoNkouatchah, Telex Magloire NgatchedCangialosi, DanieleNo, AlbertCánovas, JoseNo, J.-S.Canovas, RodrigoNoori, NavidehCao, BingyangNott, DavidCao, ChuanhaiNouri, NimaCao, JindeNovak, EdCao, ShaolongNowaczyk, SławomirCao, Wen-PingNowicki, MichałCao, YulianNozariasbmarz, AminCao, ZehongNozzoli, FrancescoCao, ZhangNtantogian, ChristoforosČapek, JanNuida, KojiCapistrano, Abraao J.S.Nunes, EricCapizzi, GiacomoNusenu, Shaddrack YawCaponetto, RiccardoO’Connell, Robert F.Caragea, CorneliaO’Kane, JasonCaraiani, PetreOdeh, Suhail M.Caravelli, FrancescoOgasawara, HaruhikoCarbone, AnnaOguchi, TakashiCárdenas, CésarO’Hare, AnthonyCardinal, PatrickOhsawa, YukioCardona-morales, OscarOjovan, MichaelCardoso, Katia ReginaOkabe, YutakaCarletti, VincenzoOkniński, AndrzejCarli, Francesca PaolaOkopinska, AnnaCarlo, GabrielOlbrich, EckehardCarmele, AlexanderOldofredi, AndreaCarmi, AvishyOliva, DiegoCarril, Jose CarbiaOliva, PietroCarroll, ThomasOlivares, FelipeCasarin, RobertoOlivares-Robles, Miguel AngelCasimiro, AntónioOliveira Panão, Miguel R.Castellano, Javier GarcíaOliveira Passos, FredericoCasteren, Jan VanOliveira, Marcos Cesar DeCastiglione, ArcangeloOliveira, Santiago Del RioCastillo-Soria, Francisco RubénOlmos-Villalba, LuisCastro, EnriqueOlsson, SimonCataliotti, Francesco SaverioOmidvarnia, AmirCatarino, AnaOneto, LucaCaţaron, AngelOnishi, VivianiCattanei, AndreaOntanon-Garcia, L.J.Cattani, AnnaOohama, YasutadaCattani, CarloOpper, ManfredCaucci, LucaOrcioni, SimoneCavalcante, Charles CasimiroOrciuoli, FrancescoCavallaro, FaustoOrdonez, GonzaloCavallaro, GiuseppeOrdoukhanian, EdwinCavazza, JacopoOrendáčová, AlžbetaCayron, CyrilOriols, XavierCazarez-Castro, Nohe R.Orosz, GyörgyCazelles, KevinOrović, IrenaCéleri, Lucas ChibebeOrtega-Delgado, BartoloméCeptureanu, Eduard GabrielOrtiz De Zárate Leira, José MaríaCerda, MauricioOrtiz De Zárate, José M.Cerqueti, RoyOsella, SilvioCerri, GiovanniOsman, ShazaliCervigón, RaquelOstoja-Starzewski, MartinCésar, Damian AscencioOsuna-Enciso, ValentínCeulemans, ArnoutOświęcimka, PawełChacón-Acosta, GuillermoOszust, MariuszChaddad, AhmadOttaviani, CarloChaillet, AntoineOu, CongjieChakraborty, NilayOuahabi, AbdeldjalilChalishajar, Dimplekumar N.Ouellet, EricChamberlin, RalphOuerdane, HenniChamoun, N.Ougolnitsky, GuennadyChan, Kwok SumOurabah, KamelChancellor, NicholasOuyang, YingkaiChander, HarishOvchinnikov, SergeyChandra, DaryusOverill, RichardChang, Chia-LinOvgun, AliChang, HangbaeOwino, Simon OderaChang, JianxiaOxenham, Andrew JChang, Jui-YungOzel, OmurChang, XiangyuOzilgen, MustafaChang, Yao-FengOzkaynak, FatihChangqing, ShenChristov, Ivan P.Charilaou, MichalisPacciani, RobertoCharisiou, NikolaosPacheco, Fernando A.L.Charleston-Villalobos, SoniaPáez-Hernández, RicardoChaturvedi, ItiPaganelli, SimoneChatzitofis, AnargyrosPaillard, DidierChaudhary, UjwalPaja, WieslawChávez, Guillermo CámaraPal, AmitangshuChavez, MarioPal, DebasishChen, BoweiPalacio, José Carlos EscobarChen, ChiachungPaladino, ElisabettaChen, Chih-HsuanPalamidessi, CatusciaChen, Chin-LingPalazzo, PierfrancescoChen, DiyiPalečková, IvetaChen, HaiguangPaleologu, ConstantinChen, HanchiPaleu, ViorelChen, HangPalomares-Salas, José CarlosChen, JinPalomba, ValeriaChen, JinyuanPamučar, DraganChen, Li-FenPanegyres, Peter K.Chen, NanPankowska, MalgorzataChen, PengPanoutsos, GeorgeChen, TingguiPansera, Bruno A.Chen, Ting-liPanzeri, StefanoChen, WeiPaolini, ChristopherChen, XiPapadakis, GiorgosChen, YanguangPapadopoulos, FragkiskosChen, Yeou-JiunnPapadopoulos, VasileiosChen, Yuan-HoPapalexiou, Simon MichaelChen, YuhuaPapana, AngelikiChen, Zhen-SongParadisi, PaoloChen, ZhiQiangPararai, MavisCheng, Szu-ChengPareschi, FabioCheng, ZeParigger, ChristianCherniha, Roman M.Park, HogeunChhiber, RohitPark, Kyewon KohChi, SienPark, NokeunChiang, ThomasPark, SangwonChiarelli, PieroPark, Seok-HwanChiementin, XavierParr, ThomasChillali, AbdelhakimParrilla, LuisChin, Cheng SiongParvan, Alexandru S.Chinnici, GaetanoParvez, ImtiazChira, CameliaPascal, RobertChiu, Linus Y.S.Paskin-Cherniavsky, AnatCho, Dong-hoPasson, OliverCho, Jin SeoPastuszak, GrzegorzCho, Myoung WonPaszto, VitCho, Young SikPatel, SaroshChodakowska, EwaPatino, LuisChoi, Hyun-HoPatriarca, RiccardoChoi, JaeyoungPauk, JolantaChoi, SunwoongPaul, TitanChoi, Tsan-MingPaulsen, HaukeChoi, Young-SeokPavan, GianniChoo, Pei LingPavić, IvicaChotorlishvili, LevanPavlicek, PavelChou, Hsi-HsirPavsič, MatejChou, RémiPayne, James E.Choudhury, SayantanPecina, PavelChow, Kwok W.Pei, SenChristoforidis, Georgios C.Pejaś, JerzyChu, Li-KangPejic Bach, MirjanaChuang, Cheng-HungPeláez-Moreno, CarmenChui, Kwok TaiPele, DanielChun, Kwok PanPelt, Daniël M.Chwedeńczuk, JanPeña, Francisco J.Ciaglia, Florio M.Peng, JieCiaramelli, ElisaPeng, ZhenmingCiavolino, EnricoPenn, AlanCicone, AntonioPennell, MatthewCiecholewski, MarcinPepelyshev, AndreyCieslak, JakubPereira, Amaro OlimpioCimato, StelvioPeres, DavidCitro, RobertaPérez Mariño, InésCiuonzo, DomenicoPerez, ArmandoCleaver, GeraldPérez-Domínguez, LuisClemente, Riccardo DiPérez-Escudero, AlfonsoClimente-Alarcon, VicentePérez-García, VicenteCoan, Travis G.Perić, ZoranCodetta Raiteri, DanielePeriša, MarkoCoelho, LuísPeshkov, IlyaCohen, AsafPeter, Adrian M.Cohen, EliahuPetit, AntoineCohen, RamiPetkoski, SpaseCoish, BillPetkova, MilenaColangelo, GianpieroPétritis, DimitriColla, ValentinaPetrov, AlbertColonius, FritzPetrova, TatianaColonnese, StefaniaPetrovska-Delacrétaz, DijanaColuccia, AngeloPetrzela, JiriComes, Calin-AdrianPettini, MarcoComesaña, PedroPham, Viet-ThanhComin, César HenriquePiacentini, FabrizioConklin, DarrellPiancastelli, LucaConley, JamisonPiccirilli, Maria CristinaConover, William JayPiepenbrink, KurtConsolini, GiuseppePierluigi, BrandoliniConstantinou, IordaniaPietronero, LucianoContreras-Reyes, Javier E.Pietrowicz, SlawomirConvertino, MatteoPietzonka, PatrickCooray, KahadawalaPilarczyk, MarcinCorda, ChristianPiltan, FarzinCorreia, SérgioPin, PaoloCorron, Ned J.Pinho, Armando J.Costa, MadalenaPinski, FrankCosta, Manuel Filipe P.C.M.Piot-Lepetit, IsabelleCosta, V.A.F.Piqueira, José Roberto CastilhoCostanzino, NicolaPirinen, PekkaCostarelli, DaniloPirinen, Tommi A.Costea, MonicaPirjol, DanCotfas, Liviu-AdrianPirogov, SergeyCoulson, MarkPirozzi, EnricaCourtade, ThomasPiskorski, JaroslawCraciunescu, TeddyPivoluska, MatejCraft, AndrewPizzolante, RaffaeleCraik, Fergus I.M.Planat, MichelCravero, CarloPlasson, RaphaelCrawford, JosephPlastina, FrancescoCremonini, MarcoPlastino, AngeloCrepeau, JohnPleimling, MichelCrespo Bofill, PedroPljonkin, AntonCrespo Mariño, Juan LuisPluvinage, Guy A.Crippa, PaoloPoel, MannesCrisan, Gloria CeraselaPoet, RonCristinel Stoica, OvidiuPołap, DawidCrosson, ElizabethPolatidis, EfthymiosCruz, PedroPolato, MirkoCruz-Hernández, CésarPoleto, CristianoCruz-Manzo, SamuelPoletti, DarioCsaba, MagyarPoliti, AntonioCuadra, Jefferson AlbertoPollak, EliCuccoli, AlessandroPollard, Blake S.Cudennec, ChristophePollner, PeterCuesta-Frau, DavidPolverino, PierpaoloCuestas, Juan CarlosPolyanskii, NikitaCui, GuotaoPonomaryov, VolodymyrCui, HuiPons, JosefinaCui, HuijuanPopelka, StanislavCulver, R. LeePopescu, DanCunha Jr, AmericoPopescu, Daniela-ElenaCunha, Ana Paula Martins Do AmaralPopkov, Yuri S.Curiac, Daniel-IoanPorporato, AmilcareCurilef, SergioPorter, ChristopherCurtis, AndrewPortes, Leonardo L.Cysarz, DirkPortesi, MarielaCzabanski, RobertPortmann, ChristopherCzachor, MarekPortugal, RenatoCzech, PiotrPorwik, PiotrCzechowski, ZbigniewPosada, EnriqueD’Angelo, GianniPosada-Quintero, Hugo FernandoDa Costa Mattos, Heraldo S.Poti, ValerioDa Veiga, Claudimar PereiraPotirakis, SteliosDabiri, ArmanPouquet, AnnickD’Abramo, GermanoPourtousi, MohammadDai, BinPovstenko, YurijDai, MimiPovstenko, YuriyDai, ZhendongPratas, DiogoDalai, MarcoPrecup, Radu-EmilDamasevicius, RobertasPręgowska, AgnieszkaD’Amico, GuglielmoPrellberg, ThomasD’Andreagiovanni, FabioPrentner, RobertDaneshkhah, AlirezaProdanov, DimiterDang, JianwuProesmans, KarelDang-Nguyen, Duc-TienProkopenko, MikhailDani, SamirPron, HervéDaniels, BryanPrůša, VítDaniusevičiute, LauraPryamikov, AndreyDann, MarkusPrzyborski, MarekDantan, AurélienPsannis, KonstantinosDapena, AdrianaPshyk, AleksandrDarbin, Olivier EricPuebla, RicardoDarmon, DavidPuentes, GracianaDarvishian, AliPuerto, Inés M. DelDas, ManoharPulkkinen, AkiDas, NarottamPuthoor, Ittoop VergheeseDas, ProdipPyo, SeungmoonDassios, IoannisQi, DiDaudaravičius, VidasQi, GuanqiuDávalos, AntonioQi, HaitaoDavid, SergioQi, YiDavila-Velderrain, JoseQian, Jin-yuanDavoudi, FatemehQian, XiaofengDe Araújo, José CarlosQiao, JunweiDe Bragança Pereira, Carlos AlbertoQiao, TongDe Campos, Luis M.Qiu, DaowenDe Castro, MarioQueiros, Silvio M. DuarteDe La Fraga, Luis G.Quevedo, HernandoDe La O Serna, José AntonioQuinet, PascalDe Leon, ManuelR Poudel, BedDe Maria, BeatriceRačkauskas, AlfredasDe Meo, PasqualeRadescu, RaduDe Novaes Pires Leite, GustavoRadhakrishnan, ChandrashekarDe Oliveira Júnior, SilvioRadivilova, Tamarade Oliveira, Leise KelliRadujković, MladenDe Saa Guerra, IvesRadziemska, MajaDe Sanctis, AngelaRafał, Marcin LaskowskiDe Santis, MicheleRagosta, MariaDe Simone, Marco ClaudioRagulskis, MinvydasDe SM Ozelim, Luan CarlosRahbar, KiyarashDe Souza, Danival JoséRahimi Tabar, Mohammad RezaDeAngelis, DonaldRajamanickam, YuvarajDębowski, ŁukaszRajashekar, RakshithDeffner, SebastianRajatheva, NandanaDeFord, DarylRajmic, PavelDehesa, Jesu’s S.Rak, RafałDehghannasiri, RoozbehRamanathan, RavishankarDeiters, UlrichRamirez, Israel ReyesDel Rio, GabrielRamirez, Pedro E.Delahaye, Jean-paulRamirez-Gonzalez, GustavoDelcea, CameliaRamirez-Reyes, AbdielDelgado-Gómez, DavidRamirez-Rojas, AlejandroDemirel, YasarRamos, ArturoDeng, JifengRamos, PedroDeng, XiaofeiRamstead, Maxwell J.D.Deng, XiaogangRangel, VictorDeng, YongRangel-Hernandez, VictorDenisova, NatalyaRangel-Magdaleno, José De JesúsDerlatka, MarcinRashidi, Mohammad MehdiDhamala, MukeshRasmussen, Jens JuulDhir, NeilRasolofondraibe, LantoDi Crescenzo, AntonioRassia, Michael (Michail) Th.Di Martino, FerdinandoRast, PhilippeDi Nardo, ElviraRathie, PushpaDiana, GiovanniRathore, ShailendraDiawara, NorouRau, A. Ravi P.Díaz Cardell, SaraRaulin, Jean-PierreDíaz-Verdejo, JesúsRauwel, ProtimaDiefenbach, DennisRauzy, AntoineDieks, DennisRaval, Devesh R.Diener, ChristianRavan, MaryamDiep, Hung T.Ravelomanana, VladyDimakopoulos, YannisRavera, EnricoDimoulas, CharalamposRayegan, RambodDing, Xiao-LiRazavi, MohsenDinis, LuisReeke, George N.Dinis, RuiRehman, Md TabishDiniz, PauloRehman, Saeed UrDionísio, AndreiaReid, DarrynDirin, AmirRemaggi, LucaDitl, PavelRempel, Erico LuizDixon, David A.Ren, JamesDixon, Matthew F.Ren, ShengbingDiykh, MohammedRen, Tsang IngDjeziri, Mohand ArabRen, XuchunDlouhý, IvoRenza, DiegoDo Nascimento, Marcelo ZanchettaRestrepo Baena, Oscar JaimeDobovišek, AndrejRestuccia, LilianaDobramysl, UlrichRevnic, CorneliaDohnal, GejzaRexha, BlerimDoja, AlbertRey, DavidDokuchaev, VyacheslavReyes-Aldasoro, Constantino CarlosDombek, GrzegorzReynaud, SergeDomínguez, Luis VergaraRhi, Seok-HoDonaldson, Ross J. DonaldsonRibeiro, Haroldo ValentinDong, WenRibeiro, RicardoDonias, MarcRice, JohnDonner, ReikRichard, Jean-MarcDos Santos, RicardoRichard, NoelDovjak, MatejaRicharte, Martín G.Downarowicz, TomaszRichman, JoshuaDowning, CharlesRichter, Wolf-dieterDrapaca, CorinaRiechers, Paul M.Drawert, BrianRini, StefanoDrissi-Habti, MonssefRios-Figueroa, Homero V.Dritselis, ChrisRipamonti, EnricoDrossos, KonstantinosRitchie, Mark E.Droz, MichelRivera, Adín RamírezDrozdz, StanislawRivera-Gómez, CarlosDrton, MathiasRivero, Cristian RodriguezDruica, ElenaRizman Zalik, KristaDu, PengyuanRizun, NinaDu, Wei-ShihRoberto, CorizzoDu, YunchengRoberts, David W.Duba, KurabachewRoberts, StephenDubey, HarishchandraRobertson, DavidDubois, Daniel M.Robertson, KatieDuch, JordiRoca, LidiaDuch, WłodzisławRocca, Mario CarlosDueck, Gerhard W.Rocha, Ana PaulaDuffy, KenRoco, José Miguel MateosDulikravich, GeorgeRodger, James A.Duncan, EarlRodrigo, AlvaroDunstan, Matthew T.Rodrigo, LlopisDutta, SoumyaRodrigues, AgathaDworak, PawełRodrigues, AlexandreDyderski, MarcinRodrigues, JoãoE., JiaqiangRodriguez, AlvaroEbaid, AbdelhalimRodriguez, JavierEckold, GötzRodriguez, NibaldoEhm, ChristianRodríguez, Rosa M.Eichhorn, MarkusRodriguez-Bravo, BlancaEid, Mohamed RabeaRodríguez-Romo, SuemiEinbeck, JochenRoga, WojciechEisencraft, MarcioRogalski, GrzegorzEl Assad, SafwanRogers, DavidEler, Danilo MedeirosRojo-Álvarez, José LuisEleuch, HichemRomagnoli, Pierre PaulElfergani, IssaRomeo, LucaEliades, MarinosRomero, JacquilineEllahi, RehmatRomero-Rochin, VictorEllerman, DavidRompante Cunha, CarlosElmogy, MohammedRondoni, LambertoElmouki, IliasRong, XiminElomaa, TapioRonquillo Lomelí, GuillermoElze, ThomasRosas, FernandoEmes, MichaelRosas-Ortiz, OscarEngelmann, SabrinaRosipal, RomanEnßlin, TorstenRoss, GordonEntin-Wohlmann, OraRossi, LucaEpicoco, ItaloRossomando, FranciscoEricson, LiselottRost, Christina M.Erkut, BurakRosu, HaretEscobar-Jiménez, RicardoRoterman, IrenaEscudero, JavierRoth, VolkerEslamimanesh, AliRothkrantz, LeonEstay, HumbertoRoumpos, ChristosEstellé, PatriceRousseau, RonaldEvangelista, Luiz RobertoRoux, Pierre-FrançoisEvans, MichaelRovelli, CarloEvans, RobinRovetta, StefanoEwees, Ahmed A.Roy, ArnabExman, IaakovRubio, HiginioFabisiak, KazimierzRubio, MyriamFabris, JúlioRucco, MatteoFacchini, AngeloRuckdeschel, PeterFaccini, FrancescoRuddell, BenjaminFaes, LucaRuess, JakobFahim, MuhammadRugh, Hans HenrikFahs, MarwanRuiz Armenteros, Antonio MiguelFaini, AndreaRuiz, Vicente GonzálezFaisal Haider, MohammadRumí, RafaelFalk, Tiago H.Ruppeiner, GeorgeFalkenburg, BrigitteRupšys, PetrasFang, XingyuanRyabko, BorisFang, YinfengRyabov, ArtemFarmer, MichaelRychtáriková, RenataFarouk, AhmedRyu, HoonFasano, GiovanniRyu, SungguenFatkullin, IbrahimRyzhov, Ilya O.Fattorini, LuigiRzazewski, KazimierzFaust, KarolineRzepecka, ZofiaFavaloro, AnthonySaadane, RachidFavela, Luis H.Saadi, IsmaïlFavini, AngeloSaarela, MirkaFavretti, MarcoSabet Jahromi, Mohammad NaserFazakas, EvaSabín, CarlosFebres, GerardoSabirov, DenisFedorov, AlexeySadeghi, ParastooFedorov, Ruslan V.Saeed, KhalidFelden, CarstenSaeedi, RamyarFelice, DomenicoSælthun, Nils RoarFernandes, Steven LawrenceSafaai, HoumanFernandez Grande, EfrenSafaei, M.R.Fernández Vázquez, EstebanSafaei, Mohammad RezaFernandez-Gamiz, UnaiSafro, IlyaFernández-Gracia, JuanSagar, Robin P.Fernandez-Lozano, CarlosSaha, AshirbaniFerracini, ChiaraSaha, BiswajitFerracuti, FrancescoSahami, AmirrezaFerrara, MassimilianoSaia, RobertoFerraro, ElenaSaid, SalemFerraz, LuísSaida, HiromiFerreira, Eduarda PintoSaika-Voivod, IvanFerreira, Joao C.Saini, RajkumarFerreira, PauloSaito, KeijiFerri, MassimoSakan, NenadFerrucci, MassimilianoSałabun, WojciechFerry, DavidSalamon, PeterFigueiras-Vidal, Aníbal R.Salas, JesúsFigueroa, Ignacio AlejandroSalazar-Pereyra, MartinFigueroa, KarinaSalk, CarlFilatrella, GiovanniSalvetr, PavelFilho, Mauro FazionSamaniuk, Joseph R.Filk, ThomasSameti, MohammadFill, Hans-GeorgSamsten, IsakFilteau, MarieSanchez Lasheras, FernandoFinn, ConorSanchez, Mauricio A.Fiorentino, MauroSánchez, René-VinicioFiori, SimoneSanchez, SalvadorFirebaugh, SamaraSandamirskaya, YuliaFiroozabadi, RezaSandoval, LeonidasFischetti, Massimo VSandoval, StevenFistul, MikhailSands, DavidFitzner, KrzysztofSANG, LiwenFlamini, FulvioSang, Tzu-HsienFlorea (Voicu), CarmenSanjuán, Miguel A.F. Floriana, GargiuloSanmartin, PaulFlouros, MichaelSantamaría, IgnacioFoa Torres, Luis E.F.Santander-Jiménez, SergioFogarty, ThomásSantoro, CorradoFont Clos, FrancescSantos, AndrésFontalvo, ArmandoSantos, Jesús DanielForman-Kay, Julie D.Santos, Jose MariaFornaro, ClaudioSantos, Marcelo FrancaFörsth, MichaelSantos, RafaelForti, MauroSantos, RubimFortuna, LuigiSantulli, GaetanoFouladirad, MitraSanz Ortiz, Ángel SantiagoFragos, VassiliosSanz Urquijo, BorjaFrancesco, FeliciSanz, EduardoFrankhauser, PierreSarabia, JoséFranzese, GiancarloSaracco, FabioFrascoli, FedericoSaracevic, Muzafer H.Fraternali, FernandoSarkar, DebajyotiFrazzica, AndreaŠarler, BožidarFredeunburg, ZacSarlis, NikolaosFreire, Juan J.Sarmiento, AuxiliadoraFresnedo, OscarSarracino, AlessandroFring, AndreasSasai, ShuntaroFriston, KarlSason, IgalFrydryszak, AndrzejSaulo, HeltonFu, ChenguangSaura, José RamónFujieda, TadashiSawerwain, MarekFukuchi, SatoshiSayood, KhalidFulcher, BenjaminSbert, MateuFÜLÖP, BAZSÓScanagatta, MauroFülöp, TamásScarfone, Antonio M.Fung, ThomasScarlett, JonathanFuster Parra, PilarScarpetta, SilviaFutrell, Richard Landy JonesScatá, MarialisaGadomski, AdamSchaller, GernotGadouleau, MaximilienScharfenaker, EllisGaggero, TomasoScharinger-Urschitz, GeorgGajic, DragoljubSchiecke, KarinGajowniczek, KrzysztofSchindlerova, KaterinaGalatolo, StefanoSchippa, LeonardoGalhano, AlexandraSchiro, MarcoGallardo-Antolín, AscensiónSchlitter, JürgenGallegos, JavierSchmandt, BastianGallegos-Funes, FranciscoSchmidt, AdamGammaitoni, LucaSchmidt, Christian AndrésGao, MichaelSchmidt, DietrichGao, PeichaoSchmitt, CorinnaGao, PengSchubert, KeithGao, ZhenzhenSchulz, Hans-JörgGarcía March, Miguel ÁngelSchumann, AndrewGarcia Navarro, PigarSchwartzman, ArminGarcia, Jesus EnriqueSchwenker, FriedhelmGarcia, MiriamSedlák, PetrGarcía-Alcaraz, Jorge LuisSeely, AndrewGarcía-Frias, JavierSeites-Rundlett, WillilamGarcia-March, Miguel A.Sekeh, Salimeh YasaeiGarcía-Massó, XavierSekhar, JainageshGarcia-Perez, ArturoSeljan, SanjaGardini, LauraSellitto, AntonioGarg, HarishSellitto, Miguel AfonsoGarrappa, RobertoSelva, MassimoGasperis, Giovanni DeSelvarajoo, KumarGatti, FabienSemenov, AlexanderGavrilik, AlexandreSemkov, KrumGavrilov, MomčiloSempi, CarloGavrilova, MarinaSenatore, RosaGavrovska, AnaSenden, MarioGay-Balmaz, FrancoisSeo, ToruGazeau, Jean-PierreSepúlveda, MauricioGe, SamSequeira Gonçalves, Paulo JorgeGeiger, BernhardSergeyev, Yaroslav D.Geisel, TheoSergi, AlessandroGençağa, DenizSerrano, JavierGeng, YanlinSerrano, José IgnacioGeorgatis, EmmanuelSerrao, CarlosGeorgiev, Danko D.Servedio, Vito D PGeorgiou, Emmanuel P.Seta, TakeshiGeorgoulis, Manolis K.Seyednasrollah, BijanGeradts, ZenoSfakianaki, EleniGerasymenko, Viktor I.Shadloo, Mostafa SafdariGerlach, MartinShahpar, ShahrokhGermano, GuidoShamshirband, ShahabGerman-Sallo, ZoltanShang, XiaopuGervino, Gianpiero P.Shang, YilunGhaffari, AbuzarShankar, EswarGhane, MahdiSharma, ManikGhannam, KhaledSharov, German SergeevichGheorghiu, VladShateyi, StanfordGherghina, Ştefan CristianShchesnovich, ValeryGheribi, Aimen GheribiShe, BingGhoraani, BehnazSheikhnejad, YahyaGhosh, IndranilSheikholeslami, MohsenGhosh, SubirShekaramiz, MohammadGhosh, SucharitaShemyakin, ArkadyGiacomini, MauroShen, ChaoGiannakeas, NikolaosShen, FeiGiannerini, SimoneSheng, BoGiannetti, NiccolòSheng, Yu-BoGianni, AretiSheremet, MikhailGiannopoulos, ApostolosSherwin, William B.Gibson, NelsonShevchuk, Igor V.Gidon, DoganShi, JieGill, Richard D.Shikano, YutakaGil-Villegas, AlejandroShimazaki, HideakiGiménez, MaiteShin, Jeong-HeonGimeno-Blanes, Francisco JavierShin, Min C.Gioia, AndreaShoaran, MahsaGiorgetti, AndreaShui, LinqiGiorgi, Gian LucaShum, KennethGiraldo, BeatrizSi, WeishengGiudici, PaoloSiami-Namin, AkbarGiuffre’, OttaviaSiddiqui, OsamahGiuliani, AlessandroSidorov, DenisGiurcaneanu, Ciprian DoruSielużycki, CezaryGiusti, AndreaSieminski, AndrzejGlorioso, PaoloSigauke, CastonGlowacz, AdamSignoles, JulienGnatyuk, SergiySilhavy, PetrGodina, RaduSilva, AdãoGoggans, Paul M.Silva, Fernando S.Gohari, Amin (Aminzadeh)Silva, IvanovitchGola, ArkadiuszSilva, Jorge F.Golak, SławomirSilva, Luiz Eduardo VirgilioGoldstein, MosheSilva-Llanca, LuisGoltsev, AlexanderSim, Jae-HoonGolubev, DmitrySimani, SilvioGomes, Heitor MuriloSimić, SrboljubGómez Aguilar, José FranciscoSimon, David S.Gomez, HectorSimon, JoanGómez-Aguilar, José FranciscoSimone, FelicettiGómez-Cuba, FelipeSimone, GabrieleGomez-Pau, AlvaroSinayski, IlyaGonca, GuvenSingh, DhananjayGonçalves, HernâniSingh, KeshavGong, PanSingh, PrashantGoniewicz, KrzysztofSingh, RajeshGonzalez Bedia, ManuelSingleton, DouglasGonzález De Santos, Luis MiguelSiniscalchi, Sabato MarcoGonzalez, HugoSitu, HaozhenGonzalez-Lopez, Veronica AndreaSivasundaram, SeenithGoo, Yeong-JiaSizochenko, NataliaGooneie, AliSK, KarthickGorban, Alexander N.Skepö, MarieGórecki, TomaszSkilling, JohnGörges, MatthiasSkoglund, MikaelGorham, CarolineSkoko, ŽeljkoGorin, ThomasSkokov, Vyatcheslav N.Górnicki, KrzysztofŠkrinjarić, TihanaGostar, AmiraliSlade, Paul F.Goyeneche, DardoSładkowski, JanGrabski, Jakub KrzysztofSledge, Isaac J.Gradojevic, NikolaSlemrod, MarshallGrasso, MatteoSluiter, Marcel H.F.Grazian, ClaraSmall, MichaelGreco, AdrianaŚmieja, MarekGrega, RobertSmirne, AndreaGregorio, Paolo DeSmith, AdamGrendar, MarianSmith, Eric P.Griffiths, AlisonSmith, RobertGrigoras, VictorSoangra, RahulGrima, CyrilSobrino, Xosé Antón VilaGrima, RamonSoekadar, SurjoGrimau Puigibert, Marcel.liSohn, InsooGriñán-Ferré, ChristianSohn, JutaeGrmela, MiroslavSohoulande, ClementGrolmusz, VinceSokolov, VadimGroza, BogdanSolcan, CarmenGrzybowska, KatarzynaSoler, Miguel ÁngelGu, YongjianSoloviev, Vladimir NikolayevichGuan, YongSoltanpur, CinnaGuaragnella, CataldoSommer, FriedrichGuariglia, EmanuelSon, JunggabGuarini, Maria RosariaSon, Ki-YoungGuazzini, AndreaSong, DongpingGudder, StanleySong, LinqiGuendelman, EduardoSong, MengjieGuerrero, JoséSong, XueyuGuerrero, PaulSonnino, GiorgioGuha, SarbariSorguven, EsraGui, WenhaoSorin, MikhailGuidi, BarbaraSørup, Hjalte Jomo DanielsenGujrati, Purushottam D.Souravlas, StavrosGullo, ParideSousa, Ana I.Gültas, MehmetSousa, Filipa L.Gunawan, IndraSousa, XulioGuney, DurduSousa-Silva, RuiGünther, ManuelSouto, AndréGuo, DezhouSouza, Andre M.C.Guo, SenSpagnolo, BernardoGuo, ShuaishuaiSpagnolo, NicolòGuo, YandongSparavigna, Amelia CarolinaGuo, YangyuSpee, CorneliaGuo, YuzhuSperlì, GiancarloGurau, VladimirSrinivasa, ArunGürcan, Özgür D.Srivastava, GautamGutiérrez, Eduardo QuevedoStace, ThomasGutiérrez-Gutiérrez, JesúsStadlbauer, ManuelGuyot, PatriceStaiano, AntoninoGuzmán Murillo, Francisco ShidarthaStan, CristinaGuzmán, LevStarace, GiuseppeHaack, GéraldineStarosolski, RomanHaber, RodolfoStathopoulos, PanagiotisHaccoun, DavidStavrou, Photios A.Hadad, YossiSteane, Andrew M.Haddad, Diego BarretoŠtefaňák, MartinHaehl, Felix M.Stefanatos, DionisisHajabdollahi, FarzanehStefanoiu, RaduHajnal, AlenStein, Bas VanHalle, BertilSteinwandt, RainerHalliwell, JonathanStepanov, NikitaHamann, MichaelStepanov, Oleg A.Hamdan, HussamStepien, KrzysztofHamed, AliSterling, MarkHammond, WilliamStern, JulioHamori, ShigeyukiStevens, DavidHamza, BenStevenson, SimonHan, GuangyueStinis, PanosHangos, KatalinStins, JohnHansen, NielsStipčević, MarioHaque, RejwanulStodola, PetrHardal, Ali Ümit CemalStojmenović, MarijaHarre, MichaelStoyanov, BorislavHarremoës, PeterStramaglia, SebastianoHarrison, WillieStratton, PeterHaruna, TaichiStreimikiene, DaliaHarvey, AllanStrekowski, RafalHashemi, Seyyed AliStrozzi, FernandaHasimoto-Beltran, RogelioStruchtrup, HenningHasrhman, Nathan LeeStrzalka, DominikHassan, Ahnaf RashikStuckey, William M.Hassan, FauziyaStudenka, BreannaHassani, BertrandSturrock, PeterHatano, NaomichiStyer, Daniel F.Hatemi-J, AbdulnasserŠtys, DaliborHatzivasilis, GeorgeSu, Wen-YuHaun, AndrewSubasi, AbdulhamitHäusler, GerdSudzina, FrantisekHaven, EmmanuelSuh, DongukHayflick, LeonardSulis, WilliamHe, Ji-huanSułowicz, MaciejHe, WangpengŠumak, BoštjanHe, WeifengSumbatov, AlexanderHe, YurongSumelka, WojciechHe, ZengyouSun, Guo-HuaHeartfield, RyanSun, HuaHébert-Dufresne, LaurentSun, Jia RuiHedayatifar, LeilaSun, JinweiHeidari, MaziarSun, KeHeinonen, MarkusSun, MeijunHenchman, RichardSun, XiaoleiHendi, Seyed HosseinSun, ZhiyongHermens, RonnieSundar, V.S.Hernández, AlfredoSuresh, Mahima AgumbeHernández, NicolásSutin, AlexanderHernández-Fernández, AntoniSvensson, Carl-MagnusHernández-Pérez, IvánSvozil, KarlHernes, MarcinSwacha, JakubHerrera-May, Agustin L.Swendsen, Robert H.Herynek, VítSyczewska, MalgorzataHeydari, JavadSzabo, AlexandreHeyden, MatthiasSzabo, LorandHigham, Desmond J.Szałowski, KarolHilbert, MartinSzámadó, SzabolcsHilbert, StefanSzavits-Nossan, JurajHiller, Sven-JürgenSzewrański, SzymonHinaut, XavierSzyc, ŁukaszHino, HideitsuSzymanowski, MariuszHiraiwa, TetsuyaSzymański, GrzegorzHirata, YoshitoTabrizi, Ali AkbarHirche, ChristophTaddei, FabioHirose, KeiTadic, BosiljkaHirsch, MatíasTaebi, AmirtahàHnilo, AlejandroTaherdoost, HamedHoang, Hong-MinhTaitel, YehudaHoblos, GhalebTajani, FrancescoHockenberry, AdamTakahashi, ShunHoel, ErikTamborrino, AntoniaHofmann, RalfTamborrino, MassimilianoHölbl, MarkoTamburini, FabrizioHolik, FedericoTame, JeremyHolmberg, HenrikTan, Syh-YuanHolst, AndersTan, VincentHolubec, ViktorTan, WeixianHomri, LazharTanaka, IsaoHong, DaesikTanaka-Ishii, KumikoHong, SungbumTang, HongHong, Wei-ChiangTang, YifaHong, WienTang, YinHooshyar, DanialTang, YingganHorák, JakubTang, Yu-HangHorng, Shih-ChengTang, Yunchao TangHoroshko, DmitriTangaro, SoniaHortal, EnriqueTantau, TillHorvát, SzabolcsTao, JinHorvath, DenisTao, JunHorvatić, DavorTapias, DiegoHorwitz, LawrenceTar, JozsefHosseini, MohsenTarabini, MarcoHossny, MohammedTarasov, Vasily E.Host-Madsen, AndersTariello, FrancescoHotta, Luiz K.Tărniceriu, DanielaHoughton, Conor J.Tarpey, ThaddeusHoujou, HirohikoTate, Shin-IchiHristov, JordanTausk, DanielHryniewicz, OlgierdTauste Campo, AdriàHsiao, BoTawfik, Abdel NasserHsiao, Hui-HsinTaylor, AndyHsiao, K.L.Taylor, Holly A.Hsieh, Cheng-HsiungTaylor, StephenHsieh, Pei-HsuanTchier, FairouzHsieh, Ping-HungTennstedt, PierreHsu, Yu-CharnTeomy, EialHu, JunlinTerebes, RomulusHu, RuifengTermini, DonatellaHu, XiaofeiTerrazas Angulo, GermanHua, ZhongyunTessarotto, MassimoHuang, GordonTestani, ClaudioHuang, Shyh-ChinTettamanzi, GiuseppeHuang, XianzhengThanh, Vo HongHuang, YishuoTheodorakis, PanagiotisHudon, NicolasThomas, GeorgeHughes, JamesThompson, JeffreyHulsman, KeesThu, KyawHumeau-Heurtier, AnneThual, SulianHuo, QinghuanTiakas, Eleftherios N.Hussain, IqtadarTian, LiyunHussanan, AbidTilloy, AntoineHwang, Jiun-RenTimothy, Jithender J.Iana, GabrielTings, BjoernIandoli, LucaTipeev, AzatIannace, GinoTithi, Jesmin JahanIasemidis, LeonTlelo-Cuautle, EstebanIbl, MartinTlili, IskanderIconaru, Simona LilianaTobar, FelipeIdrisov, EdvinTobochnik, JanIeracitano, CosimoToda, Alexis AkiraIerapetritou, MarianthiToffano, ZenoIgnaciuk, PrzemysŁawToghraee, DavoodIgual, JorgeTognoli, EmmanuelleIiyama, YutaroToher, Cormac H.Ikuta, RikizoTohme, FernandoIliev, AntonToigo, AlessandroIliyasu, AbdullahTolkacheva, Alena G.Imani, MahdiTomala, TristanImprota, GiovanniTomasiello, AlessandroIndurkhya, BipinTomasin, StefanoInuso, GiuseppinaTomczyk, KrzysztofInzillo, VincenzoTomita, Jesuino TakachiIonescu, ClaraTondi, BenedettaIovieno, MicheleTong, FengIshii, HideakiTong, Xin T.Islam, AkandToral, AntonioIspány, MártonToral, RaúlIsraeli-Korn, SimonToranzo, Irene V.Isufi, ElvinTorezzan, CristianoIsvoranu, DragosTorikai, KotaIvanov, PlamenTorres, Arnau MirIvasic-Kos, MarinaTorres, LizethIwakawa, AkiraTorres, Pedro M.B.Izquierdo, Eduardo J.Torres, PolMartinez, Genaro J.Torti, ValeriaJaber, MonaTortosa, LeandroJabrane, YounesToscano, Fernando SolerJacobs, ByronToulias, ThomasJacobs, DonaldTournaki, StavroulaJaeger, AllisonTraag, VincentJaeger, GreggTracinà, RitaJafary-Zadeh, MehdiTramontana, FabioJakóbczyk, LechTran, DatJamalabadi, Mohammad Yaghoub AbdollahzadehTranstrum, Mark K.Jamdagni, ArunaTravieso-Gonzalez, Carlos M.Jang, Han SeungTrifina, LucianJang, YunTsai, Chi-YiJanowczyk, AndrewTsai, Yuan-YuJantunen, ErkkiTsakanikas, VasilisJao, DavidTsallis, ConstantinoJasra, AjayTsao, Lung-ChuanJastrzebska, AgnieszkaTsaramirsis, GeorgiosJauregui, Juan CarlosTsau, Chun-HueiJauregui, MaxTseng, Chih-yuanJedrzejewski, ArkadiuszTsinos, Christos G.Jekova, IrenaTsiropoulou, Eirini Eleni (Greece)Jelfs, BethTsiropoulou, Eirini Eleni (USA)Jelinek, HerbertTsuchiya, NaotsuguJennings, Robert C.Tsuruyama, TatsuakiJepps, OwenTsuzuki, MarcosJesionek, MarcinTudoroiu, NicolaeJessa, MieczyslawTumuluri, UmaJia, XiaodongTuncel, ErtemJiang, LiangxiaoTuovinen, TeroJiang, XiaohongTura, JordiJiang, XueqinTuritsyn, SergeiJiao, YuboTurocy, TheodoreJimenez Del Val, IoscaniTutanescu, IonJiménez Zarco, Ana IsabelTutueva, AleksandraJiménez, Alfredo ArcosTveit, Tor-MartinJiménez-Martín, AntonioTyralis, HristosJin, Bih-YawTyrcha, JoannaJin, DiUchida, SatoshiJin, YanUgarte, Juan P.Jin, YanUl Haq, RizwanJinwoo, LeeUlhoa, Sérgio CostaJog, VarunUmbleja, KadriJoglekar, YogeshUnakafova, ValentinaJohnson, KippUnruh, DominiqueJones, ChrisUohashi, KeikoJoo, Soo-HyunUrbea, Jordi BaroJorgensen, Palle E.T.Urniezius, RenaldasJorswieck, EduardUrquiza, LuisJose, MarcoUruba, VáclavJoseph, JithinUsenko, VladyslavJou, DavidUtsumi, YasuhiroJouini, NoureddineUzhga-Rebrov, OlegJovic, AlanRibeiro, Haroldo V.Julia, StawarzVakanski, AleksandarJung, HaejoonValdes-Hernandez, AndreaJung, Ki-HyunValdivia, Juan AlejandroJung, Young-DaeValencia, José F.Juretić, DavorValencia-Cárdenas, MarisolKačur, JánValencia-Palomo, GuillermoKaczmarek, MariuszValero, AntonioKahaki, Seyed M.M.Vallianatos, FilipposKalaga, Joanna K.Vallone, GiuseppeKalogeropoulos, NikolaosValtierra-Rodriguez, MartinKam, MosheValverde-Albacete, Francisco JKaminska, DorotaValyrakis, ManousosKamińska, Joanna A.Van De Beek, JaapKanarachos, StratisVan De Velden, MichelKanavos, AndreasVan Den Ende, JefKang, JonghoonVan Der Heijden, Rens WouterKang, Suk-BokVan Der Maas, Han L.J.Kang, WeiVan Drongelen, WimKang, ZihoVan Huffel, SabineKaniadakis, GiorgioVan Hulle, MarcKao, AlbertVan Loock, PeterKapcia, Konrad JerzyVan Rossem, StevenKapitaniak, TomaszVanhaelen, QuentinKaplun, DmitriiVarando, GherardoKapuscinski, TomaszVargas, Alessandro N.Käpylä, MaaritVarotsos, P.Kara, KursatVasconcellos, Klaus L.P.Karanikas, NektariosVasko, Ivan Y.Karawia, Abdel Rahman A.Vazquez, FedericoKarayiannis, NikosVazquez-Castro, ÁngelesKarczewski, KrzysztofVecer, JanKarczmarek, PawełVega-Oliveros, Didier AugustoKarimov, ArturVeintimilla, Salvador García-AyllónKarimov, TimurVejdemo Johansson, MikaelKartci, AslihanVenhuizen, Noortje J.Karthik, P.N.Vera, TeresaKaryotis, VasileiosVerde, FrancescoKaslik, EvaVerdoolaege, GeertKastrisios, ChristosVerdú, SergioKateris, DimitriosVergara, José DavidKatira, ParagVergori, LuigiKatkov, MikhailVerma, RajkumarKatori, YuichiVersaci, MarioKatriel, JacobVezzani, AlessandroKatsakou, AntigoniVianna, Reinaldo OliveiraKatsavounis, StefanosVicente, RenatoKatz, Jonathan I.Vieira, Alex BorgesKaufman, Michael J.Vieira, Marcus FragaKaufman, MironVilenchik, DanKavic, MichaelVillani, Maria LuisaKavroudakis, DimitrisVillani, PaoloKawamoto, ShoichiVillanueva Polanco, RicardoKawan, ChristophVillecco, FrancescoKay, Jim W.Vinuesa, RicardoKazak, JanVisan, TeodorKebande, Victor RViscito, LucaKefayati, GholamrezaVitali, FrancescaKeimer, AlexanderVitanov, Nikolay K.Keirsbulck, LaurentVitányi, PaulKelbert, MarkVlahu-Gjorgievska, ElenaKeler, AndreasVo, HuyKeller, KarstenVo, TruongKellman, MichaelVogt, AndrewKeppens, ArnoVoigtmann, ThomasKeshmiri, SoheilVolkovich, ZeewKeskinarkaus, AnjaVolosencu, ConstantinKhalid, FarrukhVon Toussaint, UdoKhan, Atif AliVorwerk, JohannesKhan, GoharVoss, AndreasKhan, ImranVourdoubas, JohnKhan, Noor SaeedVozel, BenoitKhattak, Asad M.Vrbka, JaromírKhlopov, MaximVrigneau, BaptisteKhojasteh Salkuyeh, YaserVujović, IgorKhokhlov, NikolayVuorio, AlpoKhouzani, M.H.R.Waas, Anthony M.Khurshudyan, MartirosWadayama, TadashiKia, BehnamWadströmer, NiclasKida, TakuyaWagoner, JasonKiel, Lowell DouglasWakif, AbderrahimKiktenko, EvgeniyWako, HiroshiKilkovský, BohuslavWaldorp, Lourens J.Kim, AlbertWalker, AndyKim, CheonshikWallhoff, FrankKim, DonghyunWan, ThomasKim, Eun-jinWang, AnzhongKIM, Heuy DongWang, BoKim, HyunjuWang, Charlie C.L.Kim, Ji-HwanWang, ChengKim, JonghoekWang, DongKim, Jong-MinWang, GangJinKim, Man-HoeWang, GangshengKim, Min SooWang, HuaqingKim, Sang-HyoWang, JianhuiKim, SoojunWang, JiayinKim, Tai-hoonWang, JingKim, Won HwaWang, JunKing, Adam C.Wang, KaiKinsner, WitoldWang, KezeKirchhoff, Michael D.Wang, KunchingKirillov, AlexanderWang, LeiKirste, ThomasWang, LiKish, Laszlo B.Wang, Li-ChunKishore, P Venkata VijayWang, LigangKiss, Laszlo I.Wang, MinKisvarday, ZoltanWang, PingKitagawa, JiroWang, QianKiukas, JukkaWang, QiwenKiyono, KenWang, ShaohuiKleiber, ChristianWang, ShaoqingKleidis, KostasWang, ShiyanKlein, IngoWang, WeiKlika, VáclavWang, WenyiKloc, LubosWang, XiKloker, Markus J.Wang, XianzhiKnauth, StefanWang, XingyuanKnežević, GoranWang, XuewenKnizley, AltaWang, YingyingKo, Seung-WooWang, Yuan-KaiKochanek, KrzysztofWang, Yu-KaiKogias, DimitrisWang, YunKoh, JaehanWarwick, KevinKoh, Yee RuiWąs, JarosławKohno, NobuakiWatanabe, EiichiKolchinsky, ArtemyWegewijs, MaartenKoltsov, SergeiWei, BoKolyukhin, DmitriyWei, Chun-ShuKomati, RajeshWei, JinlongKondylakis, HaridimosWei, LuKong, DeguangWei, PingKong, FandeWei, Qi-HuoKoniaris, MariosWei, ShuangqingKonstantinides, DimitriosWeinberger, NirKoplenig, AlexanderWeinert, FriedelKoprinkova-Hristova, PetiaWeiss, ChristianKorbel, JanWeitschek, EmanuelKordana, SabinaWen, BaoleKordov, KrasimirWendin, GöranKorodi, AdrianWernik, JacekKorzhik, ValeryWessel, NielsKorzun, DmitryWettstein, MartinKosloff, RonnieWhitaker, AndrewKossowski, TomaszWhite, CraigKostina, VictoriaWhitney, RobertKostur, MarcinWibral, MichaelKot, SebastianWichert, AndreasKotelba, AdrianWielgosz, MaciejKotsiantis, SotirisWilson, SimonKotyński, RafałWilson, StuartKoubiti, MohammedWinkler, David A.Kouchaki, SamanehWinther, OleKoumou, Gilles BoeviWiora, JózefKouziokas, Georgios N.Wissner-Gross, AlexanderKovács, RóbertWitzany, JiříKovalenko, Ilya G.Włodarczyk, ZbigniewKovari, AttilaWohlrabe, KlausKovchegov, YevgeniyWolfer, SaschaKowalski, GregoryWollstadt, PatriciaKowalski, JerzyWong, David Shan-HillKozyrev, SergeyWong, Ka-ChunKrakovská, AnnaWong, Tzu-TsungKralj, SamoWong, Wing-KeungKrasnopolsky, Vladimir M.Woodbury, Allan D.Krasteva, VesselaWootters, WilliamKratzke, NaneWorley-Davis, LynnKrause, DécioWrzesiński, DariuszKrawec, Walter O.Wu, BoKrejcar, OndrejWu, ChenshengKretz, TobiasWu, ChunxueKrewinkel, RobertWu, Hao-TianKriksciuniene, DaliaWu, Hsien-TsaiKrishnamurthy, K.Wu, Ja-lingKristjanpoller, FredyWu, JianhuaKroesen, MaartenWu, JinsongKröker, IljaWu, JiyanKrssak, MartinWu, TailinKrstacic, GoranWu, XiaofengKrüger, BjörnWu, XiugangKrztoń-Maziopa, AnnaWu, YuefengKrzyzak, AdamWu, Yu-WeiKubicek, JanWulandana, RachmadianKudra, GrzegorzWyffels, FrancisKudryashov, BorisXavier, PradipKujala, TuomoXiao, BinKukulka, David J.Xiao, BoqiKułakowski, KrzysztofXiao, GaoxiKullie, OssamaXiao, HongKumar, DevendraXiao, ZhouKumar, KrishnaXie, DanmeiKumar, SudhirXie, HongboKumar, SuhasXie, JiafengKumar, VinayXie, ZhonghuaKumara, IndikaXie, ZuotiKumon, MakotoXu, ChiKundu, SnehasisXu, JianKunert-Graf, JamesXu, JunKung, Chien-ChunXu, MingtianKuo, WatsonXu, WeitaoKuok, Sin-ChiXu, XiangyangKuribayashi, MinoruXu, ZhipingKurnitski, JarekXue, XiaodaiKuś, MarekYaïci, WahibaKutner, RyszardYamagishi, HiromitsuKuzilek, JakubYamagishi, MasaoKuznetsov, Nikolay V.Yamaguchi, TetsuoKvålseth, Tarald O.Yamano, TakuyaKwak, Ho YoungYan, QifaKWAK, Young HoonYan, RuqiangKwapień, JarowslawYanagisawa, DaichiKwek, Leong ChuanYang, FanKwuimy, CedrickYang, GuangKycia, RadosławYang, HongwenKyriazis, Nikolaos A.Yang, Howard H.La Magna, AntoninoYang, JingweiLaBarge, Mark A.Yang, Lee-XiengLadaci, SamirYang, LicaiLaguna, HumbertoYang, SamLahoz, RafaelYang, WenchengLahoz-Beltra, R.Yang, WenxianLaín, SantiagoYang, YongjieLaity, Peter R.Yang, YuguangLake, Douglas E.Yang, YukaiLambic, DraganYao, LinaLammert, Paul E.Yarotsky, DmitryLand, MartinYazdanpanah, HamedLandi, GabrielYe, XujiongLanga, Jose A.Ye, ZhengmaoLange, HolgerYeh, Chien-HungLapidoth, AmosYeh, Rong HuaLara Molina, Fabian AndresYen, JianLardizabal, CarlosYentes, JenniferLars, Kirkby JustinYeom, Dong-hanLashin, SergeyYi, GangmanLaso, ManuelYi, Sang-HoonLaval, Jean-PhilippeYi, XinpingLawler, Michael JYin, JunLazarescu, MihaiYin, YinLazopoulos, Konstantinos A.Yokoyama, NaotoLe Bot, AlainYoo, Do GuenLe Courtois, FlorentYoon, Hyung-KooLe Rouzic, ArnaudYoon, Tae JunLe, NamTuanYordanova, KristinaLeao, JeremiasYoshida, HiroakiLee, Cheng-ChiYoun, Hee YongLee, Chyi LinYousif, CharlesLee, DerherYu, HaoranLee, HyoungsoonYu, LouisLee, JaesungYu, NingLee, JayYu, ShujianLee, JulianYu, XiaoquanLee, KeunhwaYu, YangLee, SangkyunYuan, FeiLee, Yang-hanYuan, TaoLefèvre, EricYuen, Ka-VengLeggio, BrunoYuksel, SerdarLehmann, SuneYunusa-Kaltungo, AkiluLeibovici, Didier GYurchenko, NikitaLeinonen, MarkusYusaf, TalalLeister, WolfgangYusenko, KirillLenk, PeterZaccaria, AndreaLent, Craig S.Zagrovic, BojanLenzi, Ervin K.Zaharia, Mihai HoriaLenzi, Ervin KaminskiZahorian, Stephen A.Lenzi, MarceloZaidi, AbdellatifLeonski, WiesławZak, GrzegorzLerga, JonatanZaka Ullah, MalikLerma, ClaudiaZakinyan, ArthurLesage, James P.Zakrzewski, JakubLessig, ChristianZambrano-Serrano, ErnestoLeung, CarsonZampieri, MarcosLeVine, Michael V.Zanella, MattiaLey, ChristopheZanin, MassimilianoLeyvraz, FrançoisZanjani, Mehdi B.Lhotska, LenkaZárate, Laura RosalesLi, Che-MingZárate, Luis E.Li, ChengZaslavsky, NogaLi, ChengqingZavadskas, Edmundas KazimierasLi, ChunbiaoZeevat, HenkLi, DeminZeifman, AlexanderLi, FengZeković, ZoranLi, GuanchenZelevinsky, VladimirLi, GuonengZeller, Camila BorelliLi, HaiqinZemcik, PavelLi, JingZenil, HectorLi, KevinZeydan, EnginLi, KezhiZhang, AnruLi, LongjiangZhang, ChaoLi, MinZhang, ChuanLi, MingZhang, GuannanLi, PengZhang, HanLi, ShengZhang, HaochunLi, TaiyongZhang, HuaLi, WeideZhang, JingLi, Xiao JianZhang, JixiangLi, XiaoliZhang, KaiLi, XuejunZhang, MingLi, YiZhang, QiLi, YongboZhang, QichunLi, YukunZhang, RuizhiLi, YuxingZhang, SuLi, ZhixiongZhang, XiLiang, JiyeZhang, XianzhiLiang, RuiyuZhang, Xiaohua DouglasLiang, ZhenhuZhang, Xiao-YuLiao, Yi-HungZhang, YanLiaw, PeterZhang, YanpengLiberman, MarkZhang, YongLicata, IgnazioZhang, YuLicitra, GaetanoZhang, YudongLidmar, JackZhang, ZheLillo-Saavedra, MarioZhang, ZhedongLim, Dae-EunZhang, Zi-KeLim, Ka RamZhao, JichaoLim, YutoZhao, WeihuanLima, MarkusZhao, YiLin, AijingZhao, ZuofengLin, Chun-LingZheng, JunLin, Gen-MinZheng, MinzhangLin, HongZheng, ShuLin, JunLinZheng, Tong-XingLin, Pin-HsunZheng, Wei-LongLin, Shih-ChunZherebtsov, SergeyLin, Shu-YenZhou, HaimingLin, Su-JienZhou, JunhongLin, Yi-LiZhou, MengChuLin, Yuan-PinZhu, FukangLineweaver, CharleyZhu, GuohunLing, JiazhenZhu, TianqingLinial, MichalZhu, XiaoweiLink, JasonZhu, YongLio, YuhlongZhu, YungangLipowski, AdamZiabari, AmirkoushyarLittle, Douglas JZiemkiewicz, DavidLiu, Chun-HoZierenberg, Johannesliu, DatongZiff, RobertLiu, FengshanŽilinskas, AntanasLIU, HangjinZimon, DominikLiu, I.-ChungZiouche, KatirLiu, JianqiZitikis, RicardasLiu, JingboZizzi, PaolaLiu, Keng-HaoZlatanović, IvanLiu, LiZlotnik, AlexanderLiu, QiZnojil, MiloslavLiu, Rey-LongZorica, DusanLiu, ShuaiZorzi, MattiaLiu, ShuaiqiZotin, Alexey AlexandrovichLiu, TaoZou, XiangjunLiu, TieZou, YuanchuanLiu, TongliangZsuzsanna, Onet-MarianLiu, Wei-ChengZuccon, PaoloLiu, XinfengZufiria, Pedro J.Liu, YangZunino, LucianoLiu, ZhengjunZuo, YiLiu, ZhileiŽupanović, PaškoLivieris, Ioannis E.Żur, Krzysztof KamilLizama-Pérez, Luis AdrianZurita, GroverLizier, JosephZverev, Vladimir I.Lo Franco, RosarioZyczkowski, Karol

